# Entrainment and Spike-Timing Dependent Plasticity – A Review of Proposed Mechanisms of Transcranial Alternating Current Stimulation

**DOI:** 10.3389/fnsys.2022.827353

**Published:** 2022-02-24

**Authors:** Sreekari Vogeti, Cindy Boetzel, Christoph S. Herrmann

**Affiliations:** ^1^Experimental Psychology Lab, Department of Psychology, European Medical School, Cluster for Excellence “Hearing for All”, Carl von Ossietzky University, Oldenburg, Germany; ^2^Neuroimaging Unit, European Medical School, Carl von Ossietzky University, Oldenburg, Germany; ^3^Research Center Neurosensory Science, Carl von Ossietzky University, Oldenburg, Germany

**Keywords:** transcranial alternating current stimulation, oscillations, entrainment, spike-timing dependent plasticity, STDP, non-invasive brain stimulation (NIBS), tACS

## Abstract

Specific frequency bands of neural oscillations have been correlated with a range of cognitive and behavioral effects (e.g., memory and attention). The causal role of specific frequencies may be investigated using transcranial alternating current stimulation (tACS), a non-invasive brain stimulation method. TACS involves applying a sinusoidal current between two or more electrodes attached on the scalp, above neural regions that are implicated in cognitive processes of interest. The theorized mechanisms by which tACS affects neural oscillations have implications for the exact stimulation frequency used, as well as its anticipated effects. This review outlines two main mechanisms that are thought to underlie tACS effects – entrainment, and spike-timing dependent plasticity (STDP). Entrainment suggests that the stimulated frequency synchronizes the ongoing neural oscillations, and is thought to be most effective when the stimulated frequency is at or close to the endogenous frequency of the targeted neural network. STDP suggests that stimulation leads to synaptic changes based on the timing of neuronal firing in the target neural network. According to the principles of STDP, synaptic strength is thought to increase when pre-synaptic events occur prior to post-synaptic events (referred to as long-term potentiation, LTP). Conversely, when post-synaptic events occur prior to pre-synaptic events, synapses are thought to be weakened (referred to as long-term depression, LTD). In this review, we summarize the theoretical frameworks and critically review the tACS evidence for each hypothesis. We also discuss whether each mechanism alone can account for tACS effects or whether a combined account is necessary.

## Introduction

Neural activity has been referred to as “spontaneous,” as it may be recorded from the brain seemingly without anything external inducing the activity. The largest and most synchronized activity in the brain seems to occur when people do not engage in a task, or when they are in states associated with lack of consciousness (e.g., anesthetized; Buzsáki and Draguhn, 2004; Buzsáki, 2006). Hence, neural oscillations were once thought to reflect “‘noise’ and ‘idling”’ (Buzsáki, 2006, p. 12). Since then, several studies have established that bands of oscillations are associated with a range of cognitive and behavioral functions. For example, theta has been associated with memory (Rutishauser et al., 2010); beta associated with motor performance (Espenhahn et al., 2019); and alpha associated with visual task performance (de Graaf et al., 2013). Furthermore, there are several clinical conditions that have been associated with altered neural oscillations (e.g., abnormal oscillations in schizophrenia; Uhlhaas and Singer, 2010).

Evidence for the functional relevance of oscillations comes largely from studies examining the correlation between neural oscillations and behavioral performance. Transcranial alternating current stimulation (tACS) is a form of non-invasive brain stimulation that has been proposed as a tool for selectively modulating specific neural frequencies and examining their functional relevance in a causal way (see Herrmann et al., 2016, for a discussion). The following section provides a brief overview of the method (see Herrmann et al., 2013; Vosskuhl et al., 2018, for more detailed reviews on tACS methodology).

## Brief Overview of Transcranial Alternating Current Stimulation

**FIGURE 1:**
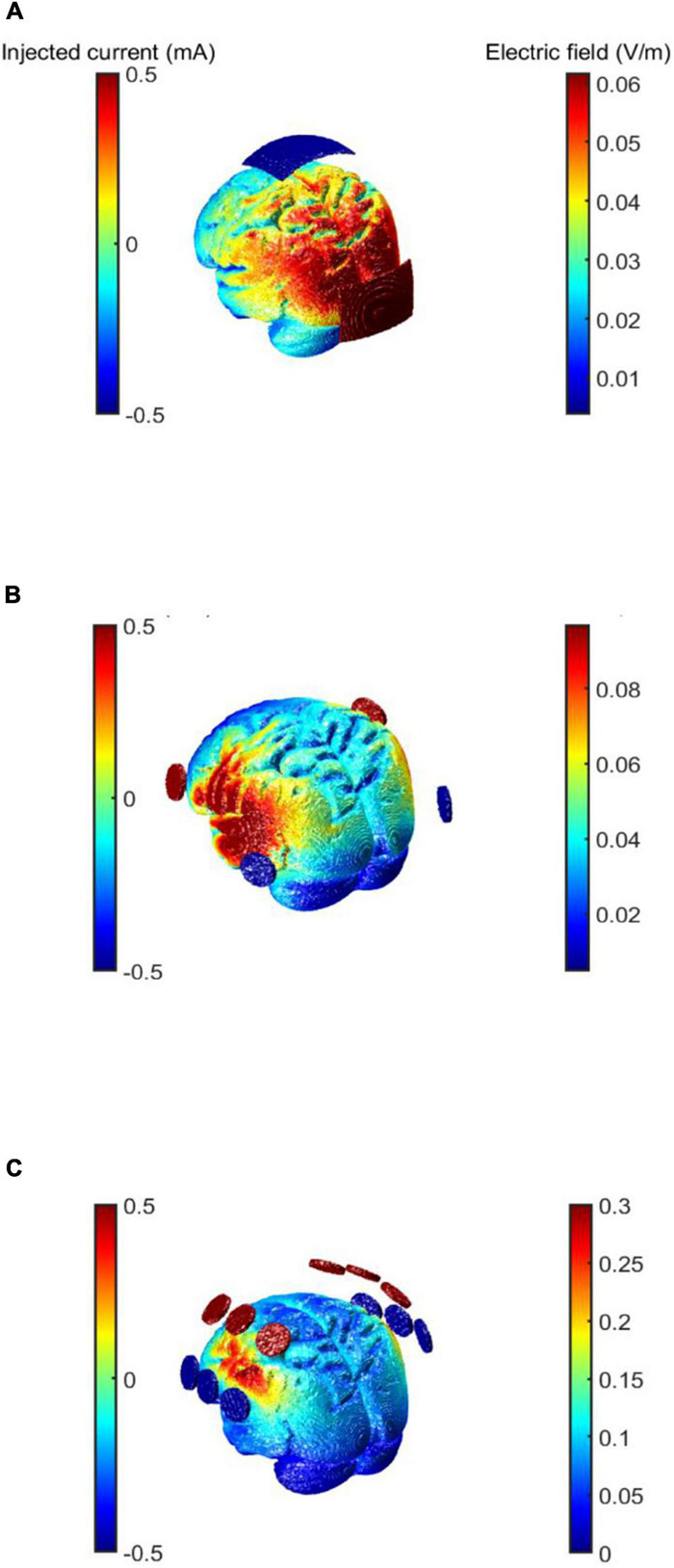
Choice of electrode montage using electric field simulations. Three different examples of tACS electrode montages to show current flow depending on number and position of electrodes. Every electrode in the figure switches between anodal and cathodal. The current intensity is 1 mA peak-to-peak. **(A)** Electrode montage for stimulating parieto-occipital brain regions. Two electrodes are placed at Cz and Oz (5 cm × 7 cm) according to the international 10–20 system. **(B)** Electrode montage for stimulating the temporal regions. The electrodes are placed at FC5 and P7 over the left hemisphere and at FC6 and P8 (Ø 26 mm) over the right hemisphere according to the international 10–10 system. **(C)** Electrode montage to stimulate the fronto-parietal cortex adapted from Popp et al. (2019). The electrodes are placed over FC3h and FC5, C3h and C5, CP3h and CP5 over the left hemisphere and at FC4h and FC6, C4h and C6, CP4h and CP6 (Ø 26mm) over the right hemisphere following the international 10–05 system.

Transcranial alternating current stimulation involves placing two or more electrodes on the scalp and applying low intensity alternating current that can modulate the electric field in the cortex (Antal et al., 2008). When two electrodes are placed on the scalp, the electrodes alternate as the anode and cathode, creating an alternating current that flows through the target region (see Figure 1A). Simulation toolboxes such as ROAST or SimNIBS^1^ which are plugins for MATLAB (The MathWorks, Inc., Natick, MA, United States) are typically used to visualize electric fields that are generated by different electrode montages, and stimulation intensities (see Figure 1).

The maximum possible current intensity in tACS depends on the electrode size and the resulting current density in the skin, which should not be higher than 0.1 mA/cm^2^ (Manual DC-STIMULATOR MC, neuroConn GmbH, Ilmenau, Germany). Electrodes with smaller surface areas will have a higher current density on the skin compared with bigger electrodes when applying the same current intensity. Therefore, the current density on the skin has to be calculated from the chosen current intensity and the size and number of electrodes.

An additional factor to consider when choosing the tACS stimulation intensity is that a large fraction of the stimulated current is shunted by the scalp so that only a small amount of the applied current reaches the brain (Wagner et al., 2007; Fröhlich, 2016). Thus, the intensity of tACS must be high enough to reach the intended neural sites for stimulation. Antal and Herrmann (2016) converted transcranial electrical stimulation intensities into intracranial voltage gradients, using head models which account for the conductance of the skin, skull, and brain tissues, and suggest that a threshold of 0.2 V/m in the target area is sufficient to modulate neural activity using tACS. This value is based on a study by Reato et al. (2010), who demonstrated that 0.2 V/m could modulate the ongoing neural activity in slices of rat hippocampus, when the stimulation frequency matched the endogenous oscillation frequency.

Stimulation duration varies widely between different studies (see Table 1). Durations of up to 45 min (±10 min) have been used with low intensity (<4 mA) stimulation (e.g., Laczó et al., 2012). Some studies have applied tACS in multiple sessions (each under 45 min, e.g., Müller et al., 2015; Ahn et al., 2019). Antal et al. (2017) explain that the current approach in the literature is to estimate the risk of the intended parameters (intensity, electrode size, stimulation duration) before application. In particular, if the intended parameters are different to those used in other experiments (e.g., higher intensities or longer durations of stimulation), researchers should provide a clear rationale for these parameters and gather safety information before using them with many human participants.

**TABLE 1:** Summary of stimulation parameters and main results of *in vivo* human tACS studies outlined in the review.

Study	Electrode montage and size	Stimulation intensity	Stimulation duration	Stimulation frequency	Method(s)	Sham included?	Main results
Ahn et al. (2019)	Cz: 5 cm × 7 cm F3, Fp1, T3 and P3: 5 cm^2^	1 mA	20 min Twice a day for 5 days	10 Hz	EEG, tDCS, auditory steady state auditory hallucinations questionnaire	Yes, between-subjects	Restored alpha power in a group of participants who received 10-Hz alpha tACS, in comparison with groups who received tDCS or no stimulation. Furthermore, the study also reported an increase in the auditory steady-state response and a decrease in auditory hallucinations in the group that received tACS versus the groups that received tDCS or no stimulation.
Antal et al. (2008)	Left motor cortex: 4 cm^2^ Contra-lateral orbit: 5 cm × 10 cm	0.4 mA	5 min	1 Hz, 10 Hz, 15 Hz, 45 Hz	TMS, Transcranial sinusoidal direct current (tSDSC), EEG Serial reaction time task	Yes, within-subjects	Improved implicit motor learning after 10 Hz alternating current only. No lasting behavioral effects after 1 h. No changes in any frequency bands in the EEG post versus pre-stimulation. No significant changes in MEP amplitudes.
Gundlach et al. (2016)	CP3/CP4: 4 cm^2^	1 mA	5 min	Individual somatosensory alpha (mu-α)	EEG, somatosensory detection task	Yes, within-subjects	Somatosensory perception thresholds were the same in the stimulation condition and sham. During mu-tACS, somatosensory detection thresholds were modulated as a function of the tACS phase.
Harada et al. (2020)	C3 and above right supra orbital 5 cm × 7 cm	1 mA	10 min	10 Hz 20 Hz	EEG, Visuomotor learning task	Yes, between-subjects	Performance on a visuomotor learning task improved after 10-Hz alpha stimulation in comparison with a 20-Hz stimulation and sham conditions. However, they did not find aftereffects for either stimulation condition.
	Study 1: Cz and Oz: 5 cm × 7 cm Study 2: 10 electrodes around P& and P8 targeting the extra-striate: Ø 1.2 cm	1 mA	2 min	10 Hz 40 Hz	EEG	Yes, within-subjects	Alpha stimulation increased phase amplitude coupling and gamma power became preferentially locked to the trough of the alpha oscillation. Gamma stimulation increased the amplitude envelope correlations, and reduced alpha power.
Helfrich et al. (2014a)	10 electrodes around P7 and P8 targeting the extra-striate visual cortices: Ø 1.2 cm	1 mA	40 min (2 min × 20 min blocks)	40 Hz	EEG, Ambiguous motion task	Yes, within-subjects	Increased coherence in the gamma frequency band for in-phase stimulation compared with anti-phase stimulation during and up to 20 min after the task. Increase in phase coherence was associated with better performance on the ambiguous motion task. Increased gamma coherence was confined to the parieto-occipital areas (i.e., coherence was location specific). In both stimulation conditions, there was a decrease in alpha power.
Helfrich et al. (2014b)	Cz and Oz 5 cm × 7 cm	1 mA	20 min	10 Hz	EEG	Yes, within-subjects	Increased power in the alpha frequency band post- and during stimulation versus pre-stimulation. Increase phase locking during stimulation.
Kasten et al. (2016)	Cz: 5 cm × 7 cm Oz: 4 cm^2^	Individually adapted 0.44–1.8 mA	20 min	IAF	EEG, vigilance task	Yes, between-subjects	Alpha power increased post-versus pre-stimulation compared to sham. Alpha power was significantly higher in the stimulation condition than the sham for 70 min. After 70 min, alpha power in the sham condition increased, and diminished the difference between the sham and stimulation groups.
Kasten et al. (2019)	Cz: 5 cm × 7 cm Oz: 4 cm^2^	1 mA	20 min	IAF	MEG, vigilance task	Yes, between-subjects	Variability of tACS aftereffects was significantly predicted by stimulation parameters of individual electric field modeling suggesting that individual stimulation protocols should be utilized.
Ketz et al. (2018)	F3/F4 Return at mastoids 5 cm^2^	1.5 mA	5 cycles at detected slow-wave frequency	Closed-loop delta (0.5–1.2 Hz)	EEG, Target detection paradigm	Yes, within-subjects	Closed-loop tACS during sleep enhanced sleep target detection accuracy post versus pre-sleep.
Kleinert et al. (2017)	F4, P4 and Cz: 5cm^2^	1 mA	26 min	5 Hz	EEG Match-to-sample task motor task	Yes, within-subjects	No differences in behavioral task performance following in- or out of-phase stimulation. No differences post-versus pre-stimulation power in the stimulation frequency band; However, alpha power decreased post- versus pre- sham condition but not the stimulation condition.
Laczó et al. (2012)	Cz: 4 cm × 7 cm Oz: 4 cm^2^	1.5 mA	45min ± 10min	40 Hz 60 Hz 80 Hz	Forced-choice detection task	Yes, within-subjects	Significantly decrease of contrast-discrimination thresholds during 60 Hz tACS, but no effect of 40 and 80 Hz stimulation
Lafleur et al. (2021)	C3 and C4 Ø 1.2 cm	1 mA	20min	10 Hz 20 Hz	EEG	Yes, within-subjects	No increase in alpha or beta power post- versus pre-stimulation after 10 and 20Hz stimulation respectively.
Lustenberger et al. (2016)	F3, F4: 3 cm^2^ Cz: 5 cm^2^	1 mA	1.5 s stimulation trains. Total duration was variable per person	12 Hz	EEG, EOG, EMG	Y, within-subjects	Closed-loop tACS selectively enhanced spindle activity. Enhanced spindle activity pre- versus post- sleep was correlated with improved motor memory in the stimulation and sham conditions.
Müller et al. (2015)	Cz and Oz 5 cm × 7 cm	Individually determined (1.51 ± 0.38 mA)	20 min over 5 consecutive days	IAF	EEG, Visual search task	Yes, between-subjects	Performance in the conjunction condition of a visual search task improved pre- versus post- stimulation in the group that received IAF stimulation versus sham. There was no significant difference in the performance in the easy or hard feature search task conditions.
Neuling et al. (2013)	Cz and Oz 5 cm × 7cm	1.5 mA	20 min	IAF	EEG, Vigilance task	Yes, between-subjects	Sustained increase in power in the alpha band in the eyes-open condition for 30 min, but not in the eyes-closed, or sham conditions.
Noury et al. (2016)	O10and CP4: Ø 1.2 cm	1 mA	20 min total (2 min × 10 min blocks)	11 Hz 62 Hz	EEG, MEG	Yes, between-subjects	tACS stimulation artifacts not only include the stimulation current but non-linear effects of heart beat and respiration. Existing stimulation artifact removal method still leave traces of the stimulation artifact.
Raco et al. (2016)	primary motor cortex: ring electrode internal Ø: 2.5 external Ø: 5 cm Pz: 5 cm × 6 cm	1 mA	∼20 min (3 min stimulation trains with 1 min break)	20 Hz	EEG, TMS, MEP	Yes, within-subjects	Phase dependent modulation of the MEP when TMS was applied in four different parts of the beta phase, suggesting that the neural state during stimulation is important for accounting for variations in MEPs.
Riecke et al. (2015)	T7 and T8: 5 cm^2^ Return electrodes: symmetrically to the left and right side of the midline: 5 cm × 7 cm	Individually determined 0.8 ± 0.1 mA	39.6 min total (4 min × 9.9 min blocks)	4Hz	Near-threshold auditory detection task	Yes within-subjects	Near threshold auditory train detection was modulated by the phase of delta stimulation.
Schwab et al. (2019)	4-in-1 montage (area at P7-PO7 and P8-PO8) Ø 1.2 cm	2 mA	13 min	10 Hz	EEG, ECG	No	Post- versus pre-stimulation, connectivity between two hemispheres at the sensor level was greatest when stimulation was in-phase between the two hemispheres, followed by jittered phase and then anti-phase. These effects decayed in the first 120 milliseconds after stimulation offset.
Stecher and Herrmann (2018)	Cz: 5 cm × 7cm Oz: 4.5 cm^2^	1 mA	38 min (1-, 3-, 5-, and 10-min blocks and reverse order)	IAF	EEG, visual vigilance task	Yes, between-subjects	No increase in alpha power post stimulation compared to sham. Follow up analysis suggests that a mismatch between stimulation frequency (IAF determined at the start of the experiment) and IAF at the end of the experiment may partially explain the lack of power enhancement.
Stecher et al. (2021)	Cz and Oz 5 cm × 7 cm	1 mA	20 min in total (150 8 s trains)	IAF (fixed) and closed-loop IAF	EEG, Visual detection task	Yes, between-subjects	Fixed IAF stimulation produced an increase in alpha power pre- versus post- stimulation compared to closed-loop IAF stimulation and sham. There was no phasic modulation of visual stimulus detection in any condition.
Strüber et al. (2015)	Cz and Oz 5 cm × 7 cm	Individually adjusted 0.76 ± 0.30 mA in IAF session 0.88 ± 0.37 mA in sham	600 1 s stimulation trains	IAF	EEG, Visual detection task	A control frequency (IAF × 3.1), within-subjects	No increase in alpha power post- versus pre-stimulation after short (1 s) trains of IAF stimulation in comparison to sham. No significant differences in performance in the visual detection task between conditions.
Vossen et al. (2015)	PO7, PO9, PO8, and PO10 5 cm × 7 cm	Individually adjusted 1.35–2 mA	22–30 min – depending on individual stimulation frequency	IAF *determined once for all 4 sessions	EEG	Yes, within-subjects	Increased alpha power post-versus pre-long stimulation (8 s) trains in comparison with short stimulation trains (3 s) and sham. Increase in alpha power occurred irrespective of phase continuity between long stimulation trains.
Wilsch et al. (2018)	T3 and T4: 4.18 cm Cz:5 cm × 7 cm	Individually adjusted	20 min per session	Speech envelope stimulation	EEG, Speech intelligibility task	Yes, within-subjects	Intelligibility of speech in noise was better when speech envelope tACS was applied in comparison with noise. 5.12 Hz sinusoidal fit described the modulation of sentence comprehension better than linear and quadratic fits. There was also a significant 5Hz peak in the average power spectrum post versus pre-stimulation.
Wischnewski et al. (2019)	C3 T7/F3/Cz/P3 Ø 1 cm	2 mA	15 min	20 Hz	EEG, TMS	No, but control condition was the placebo group	No increase in beta power or MEP amplitudes post – versus pre-stimulation for participants who received an NMDAR antagonist (to block the cellular mechanism thought to underlie LTP) in comparison to a group that received a placebo.
Zaehle et al. (2010)	PO9 and PO10 5 cm × 7 cm	Individually adjusted. 1.12 ± 0.49 mA	10 min	IAF	EEG	Yes, between-subjects	Alpha power in the centro-parietal electrodes of the EEG increased post- versus pre-stimulation in the stimulation versus the sham group.

Unlike other forms of non-invasive brain stimulation (e.g., Transcranial Magnetic Stimulation; TMS; or transcranial Direct Current Stimulation; tDCS) which change the overall polarity of the system, it is hypothesized that tACS increases or decreases the membrane potentials of the neurons in the stimulated region, making it more or less likely for depolarization or hyperpolarization to occur, respectively (Vöröslakos et al., 2018). Furthermore, tACS seems to modulate specific brain oscillations and could therefore be a powerful tool to investigate the functional relevance of specific brain oscillations (Vosskuhl et al., 2018). The unique ability of tACS to modulate oscillations could also be an advantage for clinical applications (see Elyamany et al., 2021, for a review of clinical studies utilizing tACS). It also has the benefits of being inexpensive, portable, and more tolerable than other non-invasive brain stimulation methods currently used in clinical studies (e.g., tDCS; see Matsumoto and Ugawa, 2017).

Studies utilizing tACS have shown modulation of perception in multiple sensory systems during stimulation (e.g., Helfrich et al., 2014a,b; Riecke et al., 2015; Gundlach et al., 2016; Wilsch et al., 2018). The effects during stimulation are referred to as “online effects,” while others demonstrate effects that last beyond the stimulation (referred to as “offline effects”). Two main mechanisms have been proposed to account for tACS effects – entrainment and spike-timing dependent plasticity (STDP). Entrainment refers to the synchronization of the endogenous oscillation to another driving frequency; in this review, we refer to the stimulation frequency used in tACS as the “driving frequency.” STDP refers to plastic changes that occur based on relative timing of the stimulated frequency to the endogenous frequency. In this review, we critically evaluate the empirical evidence for each of these proposed mechanisms and highlight gaps and directions for future research. An understanding of these mechanisms may help inform research design choices in future tACS studies that assess the effects of specific frequencies.

## Entrainment

Entrainment is defined as synchronization of an oscillating system to an external driving force, which coordinates the activity between rhythmic oscillations (Thut et al., 2011; Lakatos et al., 2019). The interaction between the internal oscillator and the external driving force is unidirectional; thus, only the internal oscillations are influenced by the external driving force and not vice versa. The frequency of the endogenous oscillations approximates the frequency of the external driving force until both rhythms become coupled (Lakatos et al., 2019).

Converging evidence for entrainment comes from animal and human studies that show correlations between behavioral performance and the phase of on-going neural frequencies. For example, Haegens et al. (2011) examined the relationship between the phase of alpha oscillations and neural firing in monkeys’ somatosensory, premotor, and motor regions while they performed a visuo-tactile discrimination task. The authors demonstrate that neural spiking was rhythmically related to the alpha oscillations with the highest firing rates at the peaks of the alpha cycle.

Although correlations between oscillations and functional consequences can provide important insights, it is difficult to establish the causal effects of the oscillations on performance as neural oscillations (the independent variables) are not directly manipulated. Therefore, using tACS as the external driving force can be a powerful tool to modulate specific frequencies and make causal inferences about neural and functional consequences (see Herrmann et al., 2016; Vosskuhl et al., 2018; Cabral-Calderin and Wilke, 2020, for reviews).

### Fixed Stimulation Frequency

A common approach for studying entrainment in tACS-studies is to administer tACS at a fixed frequency within the frequency band of interest for all participants of the study (e.g., 10 Hz for Alpha, and 20 Hz for Beta, as in Lafleur et al., 2021). Although all neural regions respond to all frequencies to some degree, there is evidence to suggest that specific corticothalamic networks have preferred frequencies (see Rosanova et al., 2009; Okazaki et al., 2021). Target regions for stimulation are chosen based on the regions that are a part of the functional neural network that is relevant for the research question. The preferred frequency of that region (i.e., the endogenous frequency) is chosen as the fixed stimulation frequency so that the endogenous frequency can synchronize to the applied frequency. Perceptual stimuli can then be presented at certain phases of an oscillation, and causal conclusions can be drawn based on differences in reported perception at different phases of that oscillation. Studies using this approach show that positive and negative oscillatory phase are generally associated with improved and impaired perception, respectively. For instance, Riecke et al. (2015) applied delta (4-Hz) stimulation while participants heard near-threshold click trains presented at different parts of the delta phase. They found that participants were better able to perceive click trains presented in the positive parts of the delta phase than those presented in the negative parts.

The lack of neural measures (e.g., EEG and MEG) to test the physiological effects of tACS in studies can sometimes limit interpretation of the neural basis of phasic modulation of behavior. In the ideal case, evidence for entrainment would come from studies that analyze behavioral and electrophysiological data during stimulation (which can help assess phase modulation during stimulation), as well as after stimulation (which can help assess the lasting effects of entrainment). Studies that do measure electrophysiological data during human tACS find a huge stimulation artifact which complicates data analysis. The artifact produced by tACS is difficult to filter out because the frequency of stimulation is often chosen to match the frequency of interest. Thus, if the artifact is not completely removed, it is likely that it would leave systematic noise that would also be consistent with evidence for neural entrainment. Although there have been several attempts to remove the stimulation artifact and analyze physiological data during stimulation (e.g., Helfrich et al., 2014a,b; Neuling et al., 2017), there is no consensus on whether any methods remove the stimulation artifact effectively (see Noury et al., 2016; Noury and Siegel, 2018).

Many researchers bypass the issue of stimulation artifact rejection by focusing on online behavioral effects (such as the phase dependent modulations of behavior described above) and/or offline physiological effects (referred to as “aftereffects”). Aftereffects may be, for example, an increase in post- versus pre-stimulation power (e.g., Zaehle et al., 2010; Neuling et al., 2013; Kasten et al., 2016), or an increase in phase coherence between two cortical regions (e.g., Helfrich et al., 2016; Schwab et al., 2019).

In studies that examine tACS aftereffects, there is mixed evidence for the effects of stimulation on specific oscillatory frequencies when stimulating with a fixed frequency. For example, Harada et al. (2020) found that performance on a visuomotor learning task improved after 10-Hz alpha stimulation in comparison with a 20-Hz stimulation and sham conditions. However, they did not find aftereffects for either stimulation condition which complicates the interpretation of their data. Neuling et al. (2012) found an increase in post- versus pre-stimulation alpha power when participants were stimulated with 10-Hz alpha stimulation; whereas Lafleur et al. (2021) did not find any increases in power in the alpha or beta frequency range after stimulating with 10 Hz and 20 Hz, respectively. The authors note some potential reasons for the lack of aftereffects in their study and the varying results in tACS-studies; among these reasons is the match between stimulation frequency and the peak of the individuals’ endogenous frequency. In the following section, we highlight the possible importance of matching the stimulation frequency to the endogenous frequency of the individual in order to achieve entrainment (Pikovsky et al., 2002; Ali et al., 2013).

### Individual Frequency Stimulation

Pikovsky et al. (2002) suggest that, at low stimulation intensities, external stimulation is more likely to entrain ongoing oscillations if the external frequency matches the frequency of the ongoing oscillation. For example, Negahbani et al. (2019) applied tACS at several frequencies and intensities, including the individual alpha frequency (IAF), while they measured activity in the posterior parietal cortex of awake ferrets. They found that matching the endogenous frequency with the stimulation frequency produced phase-locking of neural spikes, even at low stimulation intensities. In contrast, stimulation frequencies adjacent to the endogenous frequency required greater stimulation intensities to produce similar phase-locking of neural spikes.

**FIGURE 2:**
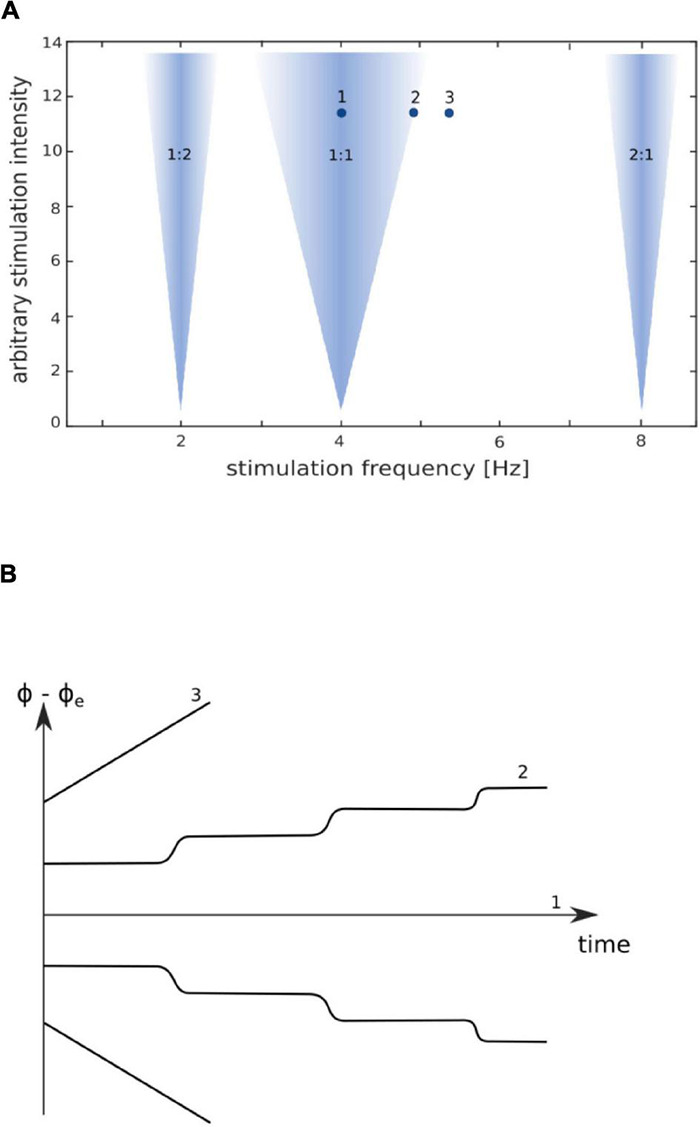
Arnold tongue with phase dynamics of synchronization adapted from Pikovsky et al. (2002). **(A)** Arnold tongue, with colored regions representing values for phase-locking between stimulation frequency and endogenous frequency. If an intrinsic oscillator is stimulated by an external driving force near its eigenfrequency (indicated by “1:1”), the external force will entrain/synchronize the endogenous oscillator to the frequency of the external driving force. Both oscillators are highly phase-locked; the phase difference is constant [indicated in **(B)** by “1”]. Greater stimulation intensities are required to synchronize the endogenous oscillator when the stimulation frequency is further from the endogenous frequency of the oscillator. This is illustrated by the wider synchronization region for higher stimulation intensities as compared with low stimulation intensities, thus forming the typical triangular shape of the Arnold tongue. Synchronization can also appear at harmonics and subharmonics of the endogenous frequency (e.g., 2:1 and 1:2). On the border of an Arnold tongue, the phase difference between external driving force and endogenous oscillation is discontinuous and alternately shows jumps and epochs with synchronous behavior [**(B)** “2”]. Further away from the border, the phase difference tends to increase uniformly [**(B)** “3”].

In contrast to animal studies, the young field of tACS research still lacks systematic human studies using an extensive range of frequencies and intensities to map out the parameter space. One approach to mapping out the parameter space is to utilize physiologically plausible computational models. For instance, Ali et al. (2013) used a large-scale cortical network model of spiking neurons to investigate the relationship between stimulation frequency and intensity of stimulation. Their model demonstrates that regions of high-synchrony between tACS and endogenous brain rhythms follow an upside-down triangular shape, referred to as the “Arnold tongue” (see Figure 2). If the stimulation intensity is very low, the frequency of the endogenous oscillator (referred to as the eigenfrequency; Veniero et al., 2015) and the applied frequency of the external driving force must be close to each other to achieve entrainment. If the stimulation intensity is higher, the external driving force could entrain the endogenous oscillations despite a mismatch between the endogenous frequency and the stimulation frequency. However, entrainment may not occur with greater mismatches between the endogenous and external frequencies. Therefore, the Arnold tongue suggests that stimulating with the individual’s endogenous frequency would be the optimal for producing entrainment.

In addition to an Arnold tongue centered on the endogenous frequency, Ali et al.’s (2013) model suggests that there are also Arnold tongues centered on the harmonics of the endogenous frequency (e.g., 1:2 or 2:1 as illustrated by the inverted triangles in Figure 2A). Furthermore, the frequency range that is entrained by the stimulation increases with higher intensity stimulation.

Supporting evidence for Ali et al.’s (2013) model also comes from several studies that correlate the size of tACS aftereffects with the match between the stimulation and endogenous frequencies. For instance, Kasten et al. (2019) showed that the match between stimulation frequency in the alpha band and IAF correlates with the size of the aftereffect. Furthermore, a mismatch between stimulation frequency and IAF over the course of the experiment also accounted for the lack of aftereffects in some conditions. Similarly, while Stecher and Herrmann (2018) reported a lack of alpha-power enhancement after stimulating participants with their IAF, when analyzing the IAF from the last observational window they found that the stimulation frequency matched the IAF in only 20 out of 44 participants by the end of the experiment. In a follow-up analysis, they found that a mismatch between the IAF and the stimulation frequency might explain the lack of tACS aftereffects in their study.

In contrast to the studies outlined above, a mismatch between the stimulation frequency and the endogenous frequency during, or at the end of, an experiment does not always account for the lack of effects observed (e.g., Ahn et al., 2019). Furthermore, a few studies have shown an inverse relationship between aftereffects and deviation between stimulation frequency and IAF (e.g., Vossen et al., 2015). However, as several parameters differ between these experiments, it is difficult to ascertain whether a mismatch between the stimulation frequency and the endogenous frequency would account for differences in aftereffects if all other parameters were equal. These mixed results highlight the need for systematic studies that specifically explore the relationship between the stimulation and endogenous frequencies and tACS effects.

### Closed-Loop Stimulation

In the previous section, we discussed how a mismatch between the endogenous frequency and the stimulation frequency may produce weaker entrainment effects. However, adding complexity to the issue, there is also evidence that the endogenous frequency within the individual shifts over time (Haegens et al., 2014; Benwell et al., 2019). Benwell et al. (2019) demonstrated that endogenous frequency determined at the start of an experiment may not match with the endogenous frequency over the entire course of the experiment. Based on the assumptions of the Arnold tongue (Ali et al., 2013) at low stimulation intensities a match between the stimulation frequency and endogenous frequency produces maximal entrainment effects. However, if the endogenous frequency is measured at the start of an experiment to determine the stimulation frequency and then the endogenous frequency shifts over time, there would be an increasing mismatch between the stimulation frequency and endogenous frequency over the course of the experiment.

In order to address the mismatch between the stimulation frequency determined at the start of the experiment and the endogenous frequency during the course of the experiment, Stecher et al. (2021) used a “closed-loop” stimulation approach. The authors compared aftereffects from a sham condition to stimulation conditions with IAF determined either at the start of the experiment, or measured and updated throughout the experiment (i.e., closed-loop stimulation). During both stimulation conditions, the authors applied 8 s of tACS, followed by 8-s breaks in which they recorded EEG. In the closed-loop condition, data from the breaks were used to calculate the IAF and update the frequency for the next 8 s of stimulation. In order to test for behavioral effects of entrainment during stimulation, participants were required to detect near-threshold light that was presented in four different phases of the stimulation sine wave.

Contrary to Stecher et al.’s (2021) hypothesis, the closed-loop protocol did not produce significantly stronger aftereffects as compared with the fixed IAF stimulation or the sham condition. However, there was a significantly stronger aftereffect for the fixed IAF stimulation condition as compared with sham. Furthermore, the authors did not find modulations of near-threshold light detection in either of the stimulation conditions compared with the sham condition. The authors suggest that this lack of behavioral modulation as a function of phase may be due to inclusion of only four bins, which Zoefel et al. (2019) suggest may be too few to observe phasic modulations given the number of trials included in the experiment. A similar study with a larger number of phase bins, or an analysis of artifact-free data during stimulation, may provide a clearer picture of whether entrainment occurred during either of the stimulation conditions.

Stecher et al. (2021) also discussed the challenges to obtaining an accurate estimate of the IAF from short recordings that are contaminated with artifacts. They suggest that future closed-loop designs may benefit from advanced online EEG artifact rejection methods, as these may allow for a better approximation of the shifting endogenous frequencies. Although their study does not provide compelling evidence for adapting the tACS stimulation frequency throughout the experiment, it highlights specific considerations and avenues for future closed-loop tACS research.

In contrast to the closed-loop approach utilized in Stecher et al. (2021), the closed-loop approach has also been used to trigger the onset of tACS based on the state of the neural system (e.g., Lustenberger et al., 2016; Ketz et al., 2018). For instance, Lustenberger et al. (2016) used the closed-loop approach to detect (in real time) transient rhythmic activity (called sleep spindles) during sleep and motor memory. The sleep spindle consisted of five peaks above the threshold of zero in the 11–16-Hz range. Once the threshold had been met, tACS stimulation was applied for 1.5 s and there was a lag of 5 s before the next threshold measurement was assessed. Using this approach, the authors found a selective enhancement in the sleep spindle activity post stimulation. Furthermore, they found an enhanced motor memory performance post- versus pre-stimulation, and this correlated with elevated spindle activity. Lustenberger et al. also found that the increase in spindle activity post- versus pre-sleep in the sham condition was also correlated with performance on the motor memory task, suggesting that there is a relationship between spindle activity and cognition. Studies such as these demonstrate the utility of a closed-loop approach, not only in tracking shifting endogenous frequencies but also in timing stimulation based on endogenous frequencies.

A similar closed-loop approach has also been implemented in the TMS literature. Here the ongoing EEG is used to trigger TMS at specific phases of the EEG (e.g., Zrenner et al., 2018; also see Zrenner et al., 2016, for a discussion of different conceptual feedback loops in TMS research). TMS and tACS have also been combined to examine phase-dependent modulation of the motor evoked potential (MEP), which is thought to be a measure of corticospinal excitability. For example, Raco et al. (2016) applied 20-Hz stimulation over the primary motor cortex and applied TMS in four different phases of the ongoing tACS (0, 90, 180, and 270). They found phase-dependent modulation of the MEP, suggesting that the neural state during stimulation is important for accounting for variations in MEPs. The results also illustrate that closed-loop tACS and TMS approaches can be promising avenues for investigating neural entrainment effects.

## Spike-Timing Dependent Plasticity

**FIGURE 3:**
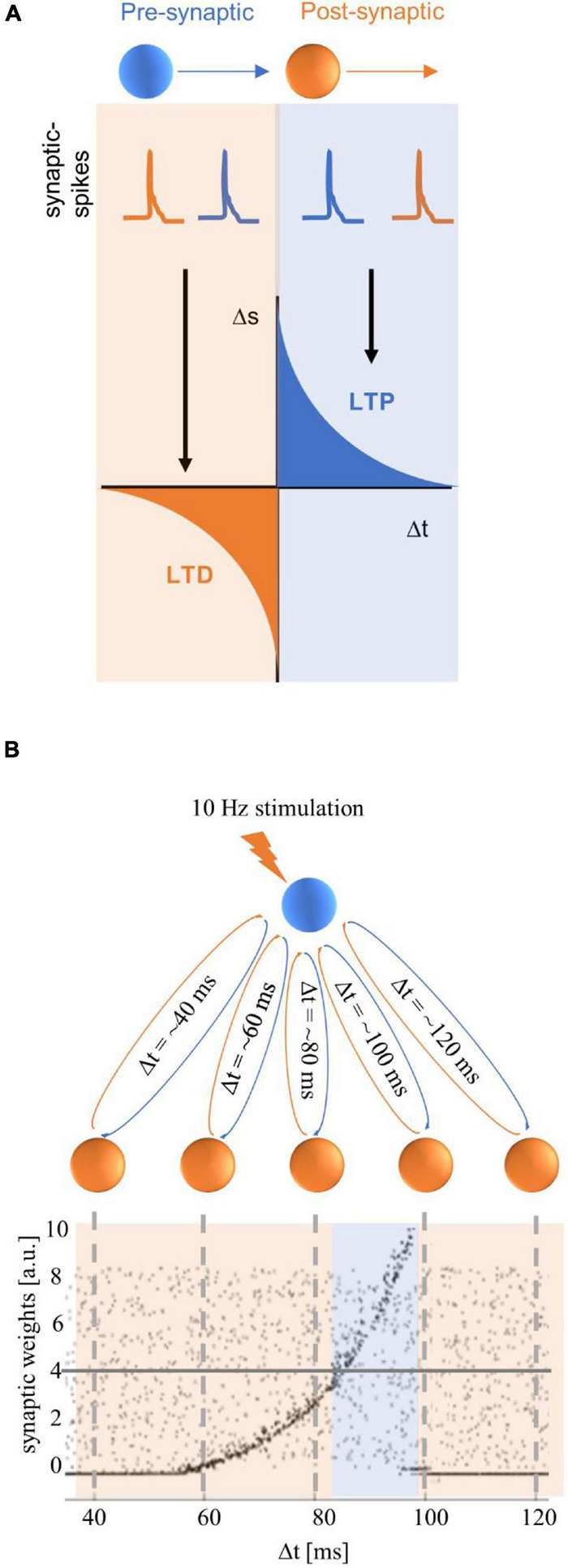
Spike-timing dependent plasticity (STDP) figures adapted from Zaehle et al. (2010). **(A)** The classic STDP curve, as described in Bi and Poo (1998), which illustrates that the size and direction of plasticity is determined by the order of pre- and post-synaptic events. The orange box illustrates an example of a post-synaptic spike (in orange) that occurs before a pre-synaptic spike (in blue), leading to a weakening of the synaptic strength that is referred to as long-term depression (LTD). The blue box illustrates an example of a pre-synaptic spike (in blue) which precedes a post-synaptic spike (in orange), leading to a strengthening of the synapse that is referred to as long-term potentiation (LTP). **(B)** A hypothetical neural network in which an excitatory driving neuron (in blue) has feedback loops with neurons in a hidden layer of the neural network (in orange). The *x*-axis on the graph shows the time taken for a complete feedback loop – or the total synaptic delay in milliseconds (Δt), and the *y*-axis shows the synaptic weights in arbitrary units (a.u.). The gray dots in the graph show the synaptic weights for recurrent loops with synaptic delays ranging from ∼40–120 ms prior to tACS. The random distribution of synaptic weights suggests that all loops have roughly equal weighting. If the driving neuron is stimulated with repetitive input (10-Hz spike trains in this example), the strength of the neural response of each loop depends on the total synaptic delay of the loop (this can also be referred to in terms of the neural resonance frequency, for example, Δt = ∼100 ms = 10-Hz resonance frequency). Post-stimulation synaptic weights are illustrated in black dots; these show that synaptic weights increased for feedback loops with resonance frequencies between ∼12 Hz (Δt = ∼80 ms) and 10 Hz (Δt = ∼100 ms). Note that although potentiation can be observed from Δt = ∼60 ms, it is only higher than the pre-stimulation baseline synaptic weights (represented by the horizontal gray line at 4 × 10^4^ synaptic weights) from Δt > 80 ms. The highest synaptic weights are observed for feedback loops with a resonance frequency that is close to the stimulation frequency (10 Hz/Δt = ∼100 ms), and synaptic weights are diminished for loops with higher resonance frequencies.

Effects that outlast tACS stimulation have also been proposed to reflect spike-timing dependent plasticity (STDP), which is dependent on the timing of synaptic events (see Figure 3A). Synaptic strength is thought to increase when pre-synaptic spikes occur prior to the post-synaptic spikes (referred to as long-term potentiation, LTP). Conversely, when post-synaptic spikes occur prior to pre-synaptic spikes, synapses are thought to be weakened (referred to as long-term depression, LTD; Bi and Poo, 1998; Dan and Poo, 2006; Caporale and Dan, 2008). STDP has been studied at multiple levels; STDP was initially examined in cell cultures, brain slices, and *in vivo*– these studies showed that the order of pre- versus post-cellular events, or neural spikes can give rise to LTP or LTD (see Dan and Poo, 2006, for detailed review).

Plasticity that appears like STDP established in animal models has also been demonstrated in the human motor cortex through the use of paired associative stimuli (PAS). For instance, Stefan et al. (2002) paired TMS stimulation with repetitive stimulation of the right medial nerve. Response to the stimulation (the MEP) was then measured via electromyography (EMG). Consistent with the principles of STDP, the MEP was potentiated when the electrical stimulation was presented before the TMS pulse, whereas the MEP was reduced when the TMS pulse was presented before the electrical stimulation. Furthermore, when the PAS procedure was conducted when participants were given a *N*-methyl-d-aspartate (NMDA) receptor antagonist that is thought to inhibit LTP, there was no potentiation of the MEP. These results suggest that STDP can be measured indirectly in humans.

Evidence for STDP in tACS studies comes from Zaehle et al. (2010), who were the first to use EEG to investigate the physiological effects of tACS. They administered IAF stimulation over the occipital cortex while recording EEG before, during, and after tACS. The authors were unable to analyze the EEG during tACS as it was contaminated with the stimulation artifact. However, analysis of the pre- and post-stimulation EEG data showed that alpha power increased significantly after stimulation in the stimulation group compared with a sham group. The authors proposed that STDP may be the underlying mechanism of the observed power difference in their experiment.

Zaehle et al. (2010) supported their STDP hypothesis with a proof-of-concept computational model (see Figure 3B). Their model consists of a single excitatory neuron with feedback connections, and feedback loops that take different durations to complete (between 20–160 ms; a subset of these durations, 40–120 ms, is illustrated in Figure 3B). When the driving neuron receives excitatory input (e.g., tACS) at 10 Hz, there are increased synaptic weights for feedback loops that take slightly less than 100 ms (illustrated by the blue box in Figure 3B). This is because a pre-synaptic “spike” from a neuron in a loop reaches the driving neuron prior to the next synaptic “spike” that is delivered from external stimulation. This model suggests that stimulation frequencies that are at or slightly lower than the resonance (or endogenous) frequency of the feedback loop would lead to synaptic strengthening (i.e., LTP); this is illustrated by the peak synaptic weights for feedback loops that take just under 100 ms in Figure 3B. If the stimulation frequency is higher than the endogenous frequency, then the post-synaptic spike on the driving neuron would arrive prior to the pre-synaptic spike from the external stimulation, and lead to a weakening of the synapse (i.e., LTD). In the model, this latter case leads to the synaptic weights summing to zero. The proposed STDP mechanism converges with effects observed in animal models which have been reviewed extensively (see Dan and Poo, 2006; Caporale and Dan, 2008; Markram et al., 2012).

Vossen et al. (2015) built on the STDP model illustrated in Zaehle et al. (2010). While Zaehle et al.’s model treats all resonance frequencies with equal weight (illustrated by the gray dots in Figure 3B), Vossen et al. (2015) explicitly incorporated the assumption that greater synaptic weights would be assigned to feedback loops with resonance frequencies corresponding to preferred frequencies of the neural network (i.e., the endogenous frequency such as the IAF). The adapted model by Vossen et al. (2015) accounts for their experimental results, which showed a positive correlation between the mismatch of the stimulation frequency (specifically, a lower stimulation frequency than the IAF) and the magnitude of the aftereffect relative to sham. However, the authors note that their proposed modified version of the STDP model remains to be systematically tested.

Wischnewski et al. (2019) were the first to provide more direct empirical support for STDP in a human tACS study. The authors gave participants either a placebo or a NMDA receptor antagonist to inhibit excitatory STDP (i.e., LTP). Participants were then stimulated with 20-Hz beta tACS targeting the motor cortex. Participants who received the placebo had an increase in power in the beta frequency band post- versus pre-stimulation, whereas participants that received the NMDA receptor antagonist did not exhibit the aftereffect. As blocking the cellular mechanism thought to underlie excitatory STDP connections seems to inhibit an aftereffect, these results could support the idea that STDP may be the underlying mechanisms of aftereffects observed in tACS studies. Wider applications of this technique may be useful to assess the role of STDP for aftereffects in frequencies other than beta.

Aftereffects have also been demonstrated across a variety of tasks and with different stimulation frequencies (see Veniero et al., 2015, for a comprehensive review of aftereffects). However, the heterogeneity of research designs across different studies can complicate and sometimes limit the interpretation of the aftereffects. For instance, Neuling et al. (2012) showed an aftereffect of alpha post- versus pre-stimulation. However, as there is evidence to suggest that alpha power naturally increases over time (Benwell et al., 2019), the aftereffect reported in Neuling et al.’s experiment may not be solely due to the tACS. The inclusion of pre- versus post-EEG measurements for a sham condition would be beneficial to examine whether observed aftereffects are due to stimulation or natural fluctuations in alpha power. Neuling et al. (2013) subsequently addressed this gap in a similar study in which they included a sham condition and administered IAF stimulation while participants had their eyes open versus closed. The authors found a sustained aftereffect in the alpha band in the eyes-open condition for 30 min, but not in the eyes-closed or sham conditions. These results may suggest the state of the network being stimulated also plays an important role on whether aftereffects are observed or not.

Most studies that report tACS aftereffects report that the effects were sustained for the duration recorded. For instance, Neuling et al. (2013) recorded EEG for 30 min after stimulation and found an increase in power in the eyes-open condition for the entire 30 min recorded. Therefore, it is uncertain how long the aftereffect lasted, and if or when it decayed. To examine the time course of aftereffects, Kasten et al. (2016) conducted an EEG-tACS study in which they continued recording EEG for 90 min after IAF stimulation. They found that alpha power was significantly higher in the stimulation condition than the sham for 70 min. After 70 min, alpha power in the sham condition increased, and diminished the difference between the sham and stimulation groups.

Similar to Kasten et al.’s (2016) finding of increased alpha power over time, Benwell et al. (2019) also reported an increase in alpha power over the course of an experiment. They measured and analyzed EEG for an hour in a visual line bisection task and in simple discrimination tasks. Participants’ IAFs reduced in frequency across time, at an average rate of 0.2 Hz per hour (and up to 2 Hz per hour within some individuals). The authors cautioned that these fluctuations, while they may seem small, are not negligible; indeed, many of the changes in frequencies that have been associated with cognitive and perceptual performance are within the range of the variations reported by Benwell et al. These fluctuations highlight the complexity of the underlying system dynamics. As the system being stimulated is not stationary, we need a more thorough understanding of the variables that interact with the effects of the stimulated frequency. Kasten et al. (2016) suggest that it would be appropriate to compare aftereffects in stimulation conditions to aftereffects in a sham condition rather than the power in a pre-stimulation block, as the latter would not account for natural increases in alpha power over time. A sham-condition comparison would certainly help unpack some of the results if the rate of fluctuation is consistent across participants. While many studies use between-subjects designs and allocate participants into stimulation and sham groups (e.g., Stecher and Herrmann, 2018), as Benwell et al. show, the fluctuations in alpha vary between individuals. Therefore, a within-subject design might provide a better comparison than between subjects. However, further studies are required to assess whether the rate of fluctuation within participants is consistent over time and across different experimental tasks.

## Are Entrainment and Spike-Timing Dependent Plasticity Mutually Exclusive?

Online effects of stimulation are typically attributed to neural entrainment (e.g., Riecke et al., 2015). However, offline effects (i.e., aftereffects) observed in tACS studies are discussed as evidence for both entrainment and STDP (e.g., Zaehle et al., 2010; Neuling et al., 2012; Vossen et al., 2015). It is unclear whether either mechanism alone can explain tACS aftereffects, or whether a combined account is necessary. The following section discusses the evidence for each of these alternatives.

### Entrainment Without Spike-Timing Dependent Plasticity

Animal studies of entrainment have shown that low intensity sinusoidal current can synchronize neuronal spiking activity to the stimulated frequency (e.g., Fröhlich and McCormick, 2010; Ozen et al., 2010), but do not produce aftereffects (see Reato et al., 2013, for discussion). Strüber et al. (2015) point out that the studies in animal models did not aim to investigate aftereffects, and suggest that the discrepancy between shorter stimulation durations in animal versus human studies may account for the lack of aftereffects. Consistent with this hypothesis, Strüber et al. did not find any aftereffects in a human EEG study using short (1-s) stimulation trains, consistent with the durations used in studies in animal models. These results converge with data from Vossen et al. (2015) who also did not find aftereffects when they applied stimulation trains of 3 s. In contrast, they did find aftereffect when they applied stimulation trains of 8 s. Taken together, these data suggest that there may be a critical duration of stimulation required to produce aftereffects.

As animal studies illustrate that entrainment is possible with short stimulation durations, it is possible that entrainment might have occurred in both Strüber et al.’s (2015) and Vossen et al.’s (2015) experiments, and that entrainment can occur without aftereffects (and by extension STDP). However, neither study presents direct evidence of entrainment (i.e., online phasic modulation of electrophysiological or behavioral data). Furthermore, Stecher et al. (2021) did not find evidence for phase-dependent modulations of near-threshold visual-stimulus detection (i.e., behavioral evidence for entrainment), despite using similar stimulation duration as (Vossen et al., 2015) (i.e., 8 s stimulation trains). Stecher et al. (2021) suggest that their results may be due to an insufficient number of phase bins utilized in the study (see Zoefel et al., 2019). Assessing online electrophysiological and/or behavioral consequences of short stimulation durations could help bridge the gap between animal studies and human studies that typically use longer stimulation durations (e.g., Neuling et al., 2012; Helfrich et al., 2014a,b). The results of these future studies would help discern whether entrainment is possible without STDP.

### Spike-Timing Dependent Plasticity Without Entrainment

In contrast to online effects of tACS, that are thought to be driven by entrainment, offline effects are discussed as if they are driven by both entrainment (e.g., Neuling et al., 2012) and STDP (e.g., Zaehle et al., 2010). This is likely due to the idea that entrainment may not disappate immediately, and thus it may be possible to observe entrainment effects for a brief period after stimulation (Halbleib et al., 2012; Hanslmayr et al., 2014).

Vossen et al. (2015) showed that entrainment may not be required to produce tACS aftereffects. They applied short durations of IAF tACS with short breaks of an equal duration. They ran four conditions: short/phase continuous (i.e., synchronized phase) with 3 s of stimulation and 3 s of break; long/phase continuous with 8 s of stimulation and 8 s of break; long/phase discontinuous (i.e., asynchronized phase) with 8 s of stimulation and 8 s of break, and phase angle changes of 0, 90, 180, or 270° between trains of stimulation; and a sham condition with only one train of stimulation at the start of the experiment.

As entrainment involves synchronization of the endogenous frequency to the stimulated frequency (which involves phase alignment), it is hypothesized that keeping the phase consistent between stimulation trains would facilitate entrainment. In contrast, changing the phase angle between stimulation trains is thought to disrupt entrainment but not STDP because changing the phase angle does not change the stimulation frequency. Vossen et al. (2015) compared pre- versus post-stimulation EEG and found a significant increase in alpha power after the long stimulation train conditions, in comparison with short stimulation trains and sham. The increased aftereffect was observed irrespective of the continuity of phase, suggesting that entrainment is not required for aftereffects.

Vossen et al. (2015) determined the IAF in the first of four sessions and used it as the stimulation frequency in all four sessions. As the individual frequencies were only determined once, Vossen et al. (2015) also examined the difference between the stimulation frequency and the IAF during each session; in most cases, the stimulation frequency was slightly below the IAF. Furthermore, the mismatch between the stimulation frequency and IAF was positively correlated with the magnitude of the aftereffect in the stimulation condition, relative to sham condition. Vossen et al.’s (2015) result suggests that a match between stimulation frequency and IAF (i.e., the endogenous frequency) may not be required to produce aftereffects (cf. Stecher and Herrmann, 2018; Kasten et al., 2019).

In another analysis, Vossen et al. (2015) compared the phase during intervals between stimulation with the phase of the stimulation train (i.e., the phase locking value), and did not find evidence for phase locking during the intervals. Lack of phase locking suggests that the effects of entrainment do not seem to last beyond the stimulation duration (assuming entrainment did occur during stimulation). Vossen et al.’s (2015) finding of aftereffects in the alpha band irrespective of phase continuity in stimulation trains, in conjunction with lack of phase locking during intervals between stimulation, seem to suggest that plasticity (perhaps STDP), and not entrainment, might be sufficient to produce tACS aftereffects.

There are also alternative interpretations of Vossen et al.’s (2015) findings. It is possible that their novel intermittent-stimulation procedure did not provide sufficient time for entrainment to occur. Most tACS studies use stimulation periods in the order of minutes (e.g., Zaehle et al., 2010; Neuling et al., 2012; Helfrich et al., 2014a,b; Kasten et al., 2016). In theory, this would provide many more oscillation cycles for entrainment to occur, which may in turn lead to aftereffects that are driven by both entrainment and STDP. However, this explanation does not undermine the finding that STDP may be sufficient to account for tACS aftereffects at short stimulation durations. A direct comparison of Vossen et al.’s (2015) results with longer continuous tACS would enable the assessment of whether entrainment could explain aftereffects using typical study durations. Furthermore, a systematic investigation of stimulation durations using (Vossen et al.’s 2015) novel paradigm could help track the time course in which phase-synchronous stimulation (and by extension entrainment) does modulate aftereffects compared with asynchronous phase stimulation.

In another line of logic, if tACS aftereffects are not associated with the specific neural oscillation, then stimulation aftereffects should be present for a wide range of frequencies (i.e., irrespective of the stimulated frequency). Kleinert et al. (2017) targeted the fronto-parietal sites of participants with bifocal 5-Hz theta stimulation that was either in-phase or anti-phase between hemispheres. They found no increase in theta power post- versus pre-stimulation. Instead, they found that alpha power decreased in the sham condition, but there was no such decrease in alpha power post- versus pre-theta stimulation. As alpha power seems to be differently modulated across individuals over time (Benwell et al., 2019), it is worth examining whether Kleinert et al.’s effects are replicable across different studies. In particular, future studies can help determine whether frequencies besides those in the alpha band also vary across time, and if they are also modulated when they are not stimulated/a part of the functional network that is stimulated.

Frequency-independent effects have also been observed when Transcranial Random Noise stimulation (tRNS), another form of non-invasive alternating current, has been applied. TRNS involves applying alternating current at a random range of frequencies and intensities (e.g., Terney et al., 2008). Ghin et al. (2021) found that tRNS applied in random frequencies ranging from 100–600 Hz produced aftereffects in the gamma frequency range. Others report that tRNS increases overall excitability, which is assessed via the magnitude of MEPs recorded post-stimulation (e.g., Chaieb et al., 2011). Antal and Herrmann (2016) suggest that tRNS effects may be attributed to modulation of ion channels and/or the noise raising the peaks of sub-threshold neural oscillations above the threshold for firing (mechanism referred to as stochastic resonance), rather than neural entrainment. Therefore, tRNS effects may support the notion STDP is possible without entrainment.

### Both Entrainment and Spike-Timing Dependent Plasticity

In contrast to stimulation effects that are unrelated to the stimulated frequency which are outlined above, if tACS aftereffects are associated with entrainment effects, they should occur only in a narrow range of frequencies (including harmonics and subharmonics, as described by Ali et al., 2013), and with frequencies that are coupled (i.e., phase synchronized; see Engel et al., 2013, for a review; also see Thut et al., 2011; Veniero et al., 2015). The majority of studies that report aftereffects have outlined frequency-specific offline effects of stimulation (e.g., Zaehle et al., 2010; Neuling et al., 2013; Kasten et al., 2016). However, there is a paucity of research in humans that systematically investigates an extensive range of stimulation frequencies and their consequences. Instead, we rely on animal research and biologically plausible neural models that can inform choices of stimulation frequency (e.g., Ali et al., 2013). Although this is a sound approach, more empirical human research investigating wider ranges of frequencies may provide strong converging evidence for frequency-specific tACS effects.

Evidence for tACS effects on cross-frequency coupling (or phase coherence) comes from studies that apply bifocal tACS over two different cortical regions, and stimulate each region with a frequency that is either in or out of phase with stimulation in the other region. For example, in an EEG study, Helfrich et al. (2014a) administered bifocal tACS targeting the parieto-occipital areas and administered 40-Hz gamma frequency stimulation which was either in-phase between the hemispheres or anti-phase (i.e., shifted by π between the hemispheres). As perception of horizontal motion is thought to be enhanced when there is phase-coupling in the parieto-occipital areas, the participants performed an ambiguous motion task during stimulation.

Helfrich et al. (2014a) used a stimulation artifact removal method and measured phase coherence between the electrodes during and after the task. They found increased coherence in the gamma frequency band for in-phase stimulation compared with anti-phase stimulation during and up to 20 min after the task. Furthermore, the increase in phase coherence was associated with better performance on the ambiguous motion task. Using source reconstruction, the authors also showed that increased gamma coherence was confined to the parieto-occipital areas (i.e., coherence was location specific). In both stimulation conditions, they also found a decrease in alpha power. Although this modulation is of a different frequency than that stimulated, it is thought to be consistent with the idea that alpha and gamma have an inverse relationship (i.e., there is phase-amplitude coupling between the two frequencies; e.g., Osipova et al., 2008; see Canolty and Knight, 2010, for a review of the functional role of cross-frequency coupling). Taken together, these results show that tACS may modulate the phase relationship between two different regions as well as phase coupling of two frequencies (e.g., alpha and gamma) that may be part of the same functional network.

Schwab et al. (2019) showed that there may be an overlap between entrainment and STDP using an alternative approach to analyzing aftereffects. They administered alpha (10-Hz) stimulation bifocally for 13 min and varied the phase relationship of the stimulated frequency between the two hemispheres. The stimulation was either in phase, antiphase, or jittered phase, where the frequency was shifted between 9.5–10.5 Hz across the two hemispheres. They analyzed the coherence of phase across the EEG sensors pre- versus post-stimulation. Pre-stimulation power was similar across all three conditions. In contrast, post stimulation, the authors reported that connectivity at the sensor level was greatest for the in-phase condition, followed by jitter, and then the anti-phase. The authors argue that connectivity differences can be attributed to the stimulation condition. However, alpha coherence decayed in the first 120 ms after stimulation offset. They also did not analyze data during stimulation. In a subsequent paper, Schwab et al. (2021) used two computational modeling methods to assess data from Schwab et al. (2019) and suggested that STDP (as described in Zaehle et al., 2010) may account for the observed effects.

Taken together, the evidence listed in the three sections above suggests that both entrainment and STDP may account for effects observed in tACS studies; however, the time course of these effects is yet to be established. There is also evidence that, at short stimulation durations, entrainment may not be required to produce tACS aftereffects (Vossen et al., 2015). A systematic investigation of the time in which in-phase versus anti-phase stimulation modulate the tACS aftereffect could help unpack the time course in which entrainment contributes to stimulation aftereffects.

In studies with humans, the choice of tACS frequency is often based on the principles of entrainment (see Pikovsky et al., 2002; and modeling by Ali et al., 2013) and stimulation based on the principles of STDP seem to be incidental. For instance, Vossen et al. (2015) intended to examine the consequences of stimulating at the endogenous frequency, as informed by the theory of entrainment, but discovered that the actual frequencies used were under the endogenous frequencies for some participants, presumably because of natural shifts in endogenous frequency (Benwell et al., 2019). Therefore, studies that explicitly investigate the consequences of stimulating frequencies slightly below and above the endogenous frequency could test whether STDP underlies tACS aftereffects.

## Conclusion

Transcranial alternating current stimulation may be a powerful tool for modulating neural oscillations and exploring their functional consequences. An understanding of the mechanisms that underlie tACS can help to inform research design choices, and guide further studies in mapping out the stimulation parameter space. In this review, we outlined two main mechanisms that have been proposed to underlie tACS effects – entrainment and STDP, and discussed whether the two accounts are mutually exclusive. In humans, online behavioral effects of stimulation are attributed to neural entrainment. Online electrophysiological data might provide more direct evidence for neural entrainment but are complicated by stimulation artifacts. Furthermore, there is no consensus about the effectiveness of stimulation artifact rejection methods. Conversely, because offline physiological effects may not require entrainment, it seems plausible that plasticity may better explain tACS aftereffects. However, evidence suggesting the absence of entrainment is not direct evidence for STDP–driven tACS aftereffects – particularly because online behavioral data are not necessarily measured in studies that assess offline physiological effects. Interpretation of the evidence (or lack thereof) for each mechanism is further complicated by the lack of standardized methods in this growing field. In this review, we have highlighted specific parameters that would benefit from more systematic investigation. Future studies that systematically assess both online and offline effects could further elucidate the independence of entrainment and STDP.

## Author Contributions

SV, CB, and CSH conceptualized the review. SV wrote the manuscript. CB and CSH wrote parts of the manuscript. All authors contributed to manuscript revision, read, and approved the submitted version.

## Conflict of Interest

CSH has filed a patent application on brain stimulation and received honoraria as editor from Elsevier Publishers, Amsterdam. The remaining authors declare that the research was conducted in the absence of any commercial or financial relationships that could be construed as a potential conflict of interest.

## Publisher’s Note

All claims expressed in this article are solely those of the authors and do not necessarily represent those of their affiliated organizations, or those of the publisher, the editors and the reviewers. Any product that may be evaluated in this article, or claim that may be made by its manufacturer, is not guaranteed or endorsed by the publisher.

## Footnotes

1
http://simnibs.org
